# Cross-sectional associations between adolescents' physical literacy, sport and exercise participation, and wellbeing

**DOI:** 10.3389/fpubh.2022.1054482

**Published:** 2023-02-28

**Authors:** Paulina S. Melby, Peter Elsborg, Peter Bentsen, Glen Nielsen

**Affiliations:** ^1^Department of Nutrition, Exercise and Sports, University of Copenhagen, Frederiksberg, Denmark; ^2^Health Promotion, Steno Diabetes Centre Copenhagen, The Capital Region of Denmark, Gentofte, Denmark; ^3^Danish School Sports, Nyborg, Denmark; ^4^Center for Clinical Research and Prevention, Copenhagen University Hospital, Bispebjerg and Frederiksberg, Frederiksberg, Denmark; ^5^Department of Geosciences and Natural Resource Management, University of Copenhagen, Frederiksberg, Denmark

**Keywords:** mental health, SEM, youth, quality of life, children, physical literacy, sport participation, exercise participation

## Abstract

**Background:**

Adolescence is a significant period in one's development of positive emotional and social wellbeing. Physical literacy (PL) is considered a determinant of physical health and wellbeing and is thought to be the foundation for an individual's engagement in physical activities. Yet, limited evidence exists on PL's association with adolescents' health and physical activity behavior. This study aims to (1) explore the associations between Danish adolescents' PL and their emotional and social wellbeing, (2) examine whether these associations are mediated by sport and exercise participation (SEP), and (3) consider if the associations differ across sex.

**Methods:**

Cross-sectional data from a national population survey were collected in 2020. The sample consisted of 1,518 Danish adolescents aged 13–15 years. PL was assessed with the validated MyPL questionnaire. The weekly time engaged in sports and exercise was self-reported. Self-esteem, life satisfaction, body satisfaction, and loneliness were measured with items from the standardized HBSC questionnaire, and a wellbeing composite score was calculated from these four measures. We constructed structural equation models with PL and sports and exercise participation as independent variables and the five aspects of wellbeing as dependent variables.

**Results:**

Positive associations were observed between PL and SEP (β = 0.33, *p* < 0.001) and between PL and the five aspects of wellbeing with β-values between 0.19 and 0.30 (*p* < 0.001). These associations were greater among girls. The association between PL and four of the five wellbeing outcomes were partly mediated by SEP with indirect effects (β) between 0.03 and 0.05.

**Conclusions:**

Results from this study support the hypotheses that PL is important for children and adolescents' wellbeing and physical activity behavior.

## Background

Adolescents' emotional and social wellbeing has been in a worrying decline over recent years (1) and is currently considered one of the greatest disease burdens among adolescents (2). Additionally, the prevalence of issues in emotional and social well-being are more common among adolescent girls compared to boys (3, 4). This is unfortunate, as adolescents' emotional and social wellbeing is crucial to their academic, cognitive, and social development (5, 6), and low wellbeing is connected to increased risks of non-communicable diseases (7, 8) and mortality (9). Wellbeing promotes mental health and alleviates related issues (2), and the World Health Organization (WHO) has declared that emotional and social wellbeing combine to form the foundation of well-functioning individuals and communities (10).

Emotional and social wellbeing, also commonly referred to as mental health (11), are associated with individual, social, and environmental factors (12), including lifestyle factors such as physical activity and sport participation (13–15). Numerous personal aspects are thought to be closely related to wellbeing, such as self-esteem (16), life satisfaction (17), body satisfaction (18), and loneliness (19). Self-esteem is defined as an individual's feelings and thoughts about their own importance and worth and is an essential part of one's self-concept (20). Self-esteem has shown to be associated with mental health in adolescence and adulthood (16). Life satisfaction is defined as an individual's cognitive appraisal of life quality from their own set of criteria (21) and is as an essential component within positive mental health (17). Body satisfaction, an aspect of body image, is defined as an individual's appraisal of their physical appearance and body based on their thoughts, feelings, and attitudes toward their body (22). Body satisfaction is seen as an element in mental health that has increased importance during adolescence (18). Loneliness is a negative feeling produced by disagreement between an individual's desired and existing social relations (23) and is associated with mental health problems (24).

Adolescence is a life-stage with increased vulnerability to mental health problems, which makes it a significant period in the development of positive mental health (25). Promoting positive mental health and preventing health problems, especially in early life-stages, is generally more effective than treating diseases (26, 27), and thus it is important to identify factors related to positive mental health in children and adolescents.

A concept that has gained increased attention for its potential in promoting physical health and wellbeing is that of physical literacy (PL) (28, 29). PL describes important individual attributes and prerequisites in engaging in and adhering to physical activities throughout life (30) and is therefore thought to be a determinant of health (31). While various definitions exist, most include the elements cardiovascular fitness, strength, motor competence, motivation, confidence, knowledge, and understanding, which are encompassed in three overall domains: physical, affective and cognitive. It has been argued that PL “can make significant contributions to quality of life” [(30), p. 32] and that higher levels of PL will lead to self-esteem, an important part of psychological well-being in physical activities (30). Further, drawing on findings in self-determination theory research, it has been previously suggested that PL could be a determinant of overall well-being (32, 33). This belief stems from the positive relation between autonomous motivation and contextual wellbeing (34), which both are strengthened by the perception of competences (i.e., the PL element of confidence), and from the fact that wellbeing in physical activities can transfer to other contexts (35) and may also transfer to overall wellbeing (36). Two recent studies have found positive correlations between PL and aspects of mental health in children and young adolescents (32, 33).

PL is thought to lay the foundation of engagement in sports and other physical activities (30, 31) that can positively affect children's and adolescents' wellbeing (13–15). A recent systematic review found that the extant evidence demonstrates a positive association between PL and physical activity (37), with emerging longitudinal evidence supporting the assumption that PL is important for physical activity later in life (37, 38). However, most studies have investigated PL and its associations with health and physical activity among children up to the age of 12 years, with only a few studies focusing on adolescents (39) and young adults (40). These studies observed similar associations as those found among children.

**Figure 1:**
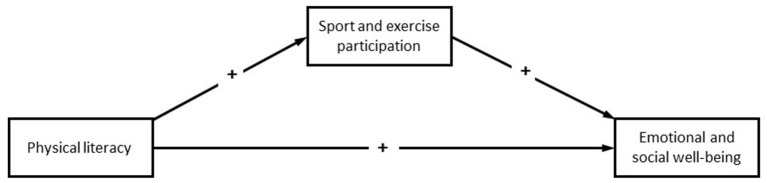
Hypothesized associations between study variables. This figure illustrates the theorized structural equation model, with aspects of emotional and social wellbeing serving as the outcomes.

Therefore, our objectives are to (a) investigate the associations between PL and aspects of emotional and social wellbeing among adolescents aged 13–15 years, (b) explore to what degree these associations are mediated by sport and exercise participation (SEP), and (c) investigate how these associations differ among boys and girls. We hypothesized that adolescents' PL would be associated with their SEP and their wellbeing and that the relationship would differ between the sexes. We further hypothesized that the relationship between PL and aspects of well-being would be partly mediated by SEP (see the hypothesized paths in Figure 1).

## Methods

### Study population

Our data came from a large-scale national survey conducted between October 29 and December 21, 2020 by Rambøll Management Consulting for the Danish Institute for Sports Studies (41). The sample of adolescents aged 13–15 years old was randomly drawn by the Danish Health Data Authority.

A slightly different questionnaire was sent to the age-groups 7–12 years old and 13–15 years old. An invitation with a weblink to the online survey was sent *via* digital mail to the parents/guardians of 9,000 children and adolescents aged 7–15 years. Two reminders were sent *via* parents/guardians' digital mail to the adolescents who had not yet completed the survey. In parallel, telephone follow-ups with the parents/guardians were conducted, encouraging the adolescents and children to participate in the survey. During the call and in the e-mails, parents/guardians could also provide the adolescents or children's private e-mail address, allowing Rambøll Management Consulting to send the invitation directly to the adolescent or child. The survey links were accessible for ~2 months. By then, 4,379 children and adolescents aged 7–15 years (48.7 % of those invited) had completed the survey, of which 1,518 were adolescents aged 13–15 years and thus included for analysis in this study. All completed answers had full data.

### Measurements

#### Measurement of physical literacy

We measured PL with the MyPL questionnaire, a context-specific questionnaire suitable for population survey, developed by the authors of this study and validated in the same sample of this study. The MyPL is a PL assessment tool that strives to account for how PL differs across different social and physical environments for physical activity, as described in the conceptualization by Whitehead (30), and to ensure that PL items will be interpreted similarly across respondents, compared to other PL assessment tools wherein participants are probed on their generic relationship toward physical activity. Confirmatory factor analysis of the model showed good fit indices (CFI = 0.938; TLI = 0.925; RMSEA = 0.065 (90% CI 0.062–0.068); SRMR = 0.055). The MyPL also showed good internal consistency and reliability for the total PL scale was 0.778 (Cronbach's alpha) and 0.783 (McDonald's Omega). The results of development and initial validation of the MyPL questionnaire is unfolded in a study be Elsborg et al. entitled “From global domains to physical activity environments: development and initial validation of a questionnaire-based physical literacy measure designed for large-scale population surveys,” which is prepared for submission. The questionnaire items and responds methods can be found in Appendix 1.

The 21-item PL scale consisted of 5 subscales: a PL for ball- and running-based activities (7 items), which consist of the elements autonomous motivation and confidence for ball and running activities combined with the physical competences of ball skills, endurance, and strength; a PL for playground-based activities (5 items) consisting of autonomous motivation and confidence for skating and climbing activities, as well as the physical competence of balance; a PL for gymnastic-based activities (4 items) consisting of autonomous motivation and confidence for gymnastics, along with physical competences for gymnastic and skipping; a PL for water-based activities (3 items) consisting of autonomous motivation and confidence for water activities combined with physical competences for swimming; and a general (not environment-specific) knowledge and understanding PL domain [3 item from the CAPL-2 (42)], which consisted of knowledge about the transfer of skills between different sports, knowledge about the importance of daily physical activity, and conceptual knowledge of strength and health.

#### Measurement of sport and exercise participation

Weekly time spent on SEP was measured with the question “How many hours do you normally use on sport/exercise per week (not counting time used on transportation)?” Participants typed in hours and minutes. Answers above 20 h were not included to minimize the risk of participants mistaking hours with minutes.

#### Measurement of aspects of mental health

##### Self-esteem

We assessed self-esteem with three items measuring participants' conceptions of others' thoughts about them and their positive self-conceptions. The participants responded to the prompts “I like myself,” “I am good enough as I am,” and “Others my age like me” using a five-point Likert scale from strongly agree to strongly disagree. The self-esteem score was calculated as the mean of the three items. The three-item self-esteem scale has showed good reliability (α = 0.89) in similar population (43) and is used in the standardized HBSC questionnaire (44).

##### Life satisfaction

We assessed life satisfaction with the Cantril Ladder (45), which is based on the above definition. Participants were presented with a ladder from zero to ten and asked to indicate “Where on the ladder do you feel you stand at the moment?” with zero indicating the worst possible life and 10 indicating the best possible (46). The Cantril Ladder has demonstrated good reliability and convergent validity (45) and is widely used, such as in the HBSC study [e.g., (47)]. Furthermore, it has shown to be related to psychological wellbeing, mood, emotions, and self-perception (48) and thus seems to be a suitable indicator of life satisfaction among adolescents.

##### Body satisfaction

We measured body satisfaction with a single item from the Body Investment Scale (49), which reflects the above definition. Participants are asked “How satisfied are you with your body (physical appearance)?” and using a 5-point Likert scale from very dissatisfied to very satisfied.

##### Loneliness

We measured global loneliness with a single item. Participants responded to the question “Do you feel lonely?” using a four-point Likert scale from “Yes, very often” to “No.” A high score reflects minor to no feelings of loneliness and is thus a positive emotional health indicator. The single-item measure of global loneliness has shown a significant relationship with the UCLA Loneliness Scale, which is an indirect multi-item scale to measure loneliness (19).

##### Wellbeing composite score

To better compare results to other studies, we decided to use a wellbeing composite score, which is the mean of the self-esteem scale (the mean of the three items) and the three single-item scores for life satisfaction, body satisfaction, and loneliness.

### Data analysis

Descriptive statistics and reliability coefficients were calculated in SPSS 25.0 (IBM Corp, Armonk, NY, USA). We used Cronbach's alpha and McDonald's omega (50) to examine the reliability of the psychometric subscales and combined scales. We considered values above 0.7 acceptable (51). For scales measuring psychological constructs with fewer than five items (i.e., self-esteem and mental health), values above 0.6 were considered acceptable (52). The values of all variables were normalized into a zero to one range to avoid high variation in the structural equation models (SEMs).

We used R studio and the lavaan packages (53) to perform an SEM with each of the aspects of wellbeing as the outcome and PL and SEP as the predictor and mediator, respectively (see the hypothesized model in Figure 1). We adjusted all models for age, and the models with the total sample were also adjusted for sex. We allowed all exogenous variables to covariate. To estimate missing values, we applied a maximum-likelihood estimation with robust standard error (MLR) values. Study variables were normally distributed (see Skewness and Kurtosis in Table 1). To inspect the model-fit indexes, we followed recommended cut-off criteria: the Tucker-Lewis index (TLI > 0.95), the comparative fit index (CFI > 0.95), and the root mean square error of approximation (RMSEA < 0.06) (54). Significance tests were two-tailed, and we considered *P*-values below 0.05 statistically significant. We only report standardized coefficients.

**Table 1:** Sample descriptive and scale reliability.

	**Mean**	**SD**	**Min**.	**Max**.	**Skewness**	**Kurtosis**	**α**	**Ω**
Competitive activities (7 items)	0.62	0.18	0.00	1.00	−0.28	−0.33	0.78	0.79
Playground activities (5 items)	0.55	0.18	0.00	1.00	−0.20	−0.19	0.68	0.68
Gymnastic-based activities (3 items)	0.48	0.27	0.00	1.00	0.14	−0.89	0.78	0.78
Water-based activities (3 items)	0.63	0.22	0.00	1.00	−0.39	−0.37	0.66	0.66
Cognitive domain (3 items)	0.75	0.23	0.00	1.00	−0.46	−0.45	0.06	0.54
Physical literacy (21 items)	0.60	0.12	0.13	0.92	−0.27	−0.09	0.84	0.73
SEP (hours/week)	5.26	3.49	0.00	19.00	1.03	0.82		
Life satisfaction (1 item)	0.73	0.17	0.00	1.00	−0.83	0.71		
Body satisfaction (1 item)	0.66	0.23	0.00	1.00	−0.58	0.17		
Loneliness (1 item)	0.85	0.22	0.00	1.00	−1.48	2.12		
Self-esteem (3 items)	0.72	0.20	0.00	1.00	−0.89	1.11	0.86	0.87
Wellbeing composite (6 items)	0.74	0.16	0.17	1.00	−0.83	0.66	0.75	0.76

Min, Minimum; Max, Maximum; SD, Standard deviation; α, Cronbach's alpha; Ω, Omega (ML).

## Results

### Descriptive statistics

The sample size was 1,518, with 51.3% being girls and a mean age of 14 years. The mean scores, standard deviations, minimum, maximum, skewness, and kurtosis for all scales and variables are reported in Table 1.

### Reliability

The internal consistency of the scales where evaluated with Cronbach's alpha and McDonald's omega (55) and are presented in Table 1. The reliability coefficients for the mental health and self-esteem scale were all above our minimum requirements. Reliability coefficients for the PL scale and the PL subscales were acceptable to good, except for the cognitive domain, where Ω- was below acceptable and α was almost zero.

### Association between physical literacy, sports and exercise participation, and aspects of wellbeing

The unadjusted intercorrelations (Pearson's R or r) among all study variables are presented in Table 2. In the total sample, PL correlated with SEP (r = 0.29, *p* < 0.001) and with all wellbeing outcomes with r-values between 0.14 and 0.20. SEP correlated with wellbeing outcomes with r-values between 0.07 and 0.14.

**Table 2:** Variable intercorrelation matrix (Pearson's R).

		**1**	**2**	**3**	**4**	**5**	**6**	**7**
1. Age								
2. Physical literacy		**−0.09**						
	Boys	−0.08*						
	Girls	**−0.11**						
3. SEP		0.03	**0.29**					
	Boys	0.06	**0.26**					
	Girls	0.00	**0.33**					
4. Life satisfaction		**−0.07**	**0.16**	**0.14**				
	Boys	−0.06	**0.11**	**0.11**				
	Girls	−0.09*	**0.23**	**0.16**				
5. Body satisfaction		−0.07*	**0.17**	**0.09**	**0.47**			
	Boys	−0.08*	**0.17**	0.08*	**0.38**			
	Girls	−0.06	**0.20**	0.08*	**0.50**			
6. Loneliness		**−0.07**	**0.14**	**0.07**	**0.46**	**0.31**		
	Boys	−0.08*	**0.12**	0.07	**0.42**	**0.21**		
	Girls	−0.07	**0.17**	0.07	**0.46**	**0.33**		
7. Self-esteem		−0.03	**0.15**	**0.09**	**0.49**	**0.53**	**0.41**	
	Boys	−0.03	**0.11**	0.04	**0.43**	**0.42**	**0.35**	
	Girls	−0.03	**0.21**	**0.11**	**0.51**	**0.58**	**0.42**	
8. Wellbeing composite		**−0.08**	**0.20**	**0.12**	**0.77**	**0.77**	**0.72**	**0.79**
	Boys	−0.09*	**0.18**	**0.10**	**0.74**	**0.70**	**0.70**	**0.76**
	Girls	−0.08*	**0.26**	**0.13**	**0.77**	**0.80**	**0.72**	**0.81**

Bold text indicates a p-value under 0.01; ^*^ indicates a p-value under 0.05.

SEMs were conducted for each wellbeing outcome—life-satisfaction, body satisfaction, loneliness, self-esteem, and the wellbeing composite score—and performed by total sample separately for boys and girls. The standardized regression coefficients (β), standard error (SE), and *p*-values for each of the models are presented in Table 3. The path from PL to SEP is included in all models. All models showed good fits (for all five models: CFI = 1.000, TLI = 1.000, and RMSEA = 0.000).

**Table 3:** Regression coefficients for the models with aspects of emotional mental health as outcomes.

	**All**			**Boys**			**Girls**		
**Paths**	**Std B**	**SE**	***P***	**Std B**	**SE**	***P***	**Std B**	**SE**	***P***
PL → Sport (all models)	0.33	0.03	0.000	0.32	0.04	0.000	0.34	0.04	0.000
**1. Wellbeing composite score**									
PL → Wellbeing (direct)	0.24	0.03	0.000	0.16	0.05	0.000	0.31	0.05	0.000
Sport → Mental health	0.10	0.03	0.006	0.09	0.04	0.011	0.10	0.04	0.016
Indirect effect	0.03	0.01	0.001	0.03	0.01	0.013	0.03	0.02	0.020
Total effect	0.27	0.03	0.000	0.19	0.04	0.000	0.34	0.05	0.000
**2. Life satisfaction**									
PL → Life satisfaction (direct)	0.19	0.04	0.000	0.10	0.05	0.042	0.27	0.06	0.000
Sport → Life satisfaction	0.12	0.03	0.000	0.10	0.04	0.000	0.14	0.04	0.001
Indirect effect	0.04	0.01	0.000	0.03	0.01	0.020	0.05	0.02	0.003
Total effect	0.23	0.04	0.000	0.14	0.05	0.006	0.32	0.05	0.000
**3. Loneliness**									
PL → Loneliness (direct)	0.23	0.05	0.000	0.16	0.07	0.022	0.29	0.08	0.000
Sport → Loneliness	0.09	0.04	0.019	0.10	0.05	0.050	0.08	0.06	0.167
Indirect effect	0.03	0.01	0.021	0.03	0.02	0.053	0.03	0.02	0.170
Total effect	0.26	0.05	0.000	0.19	0.07	0.005	0.31	0.07	0.000
**4. Body satisfaction**									
PL → Body satisfaction (direct)	0.30	0.05	0.000	0.23	0.07	0.000	0.36	0.08	0.000
Sport → Body satisfaction	0.10	0.04	0.014	0.13	0.05	0.014	0.08	0.06	0.224
Indirect effect	0.03	0.01	0.015	0.04	0.02	0.016	0.03	0.02	0.227
Total effect	0.33	0.05	0.000	0.27	0.06	0.000	0.39	0.07	0.000
**5. Self-esteem**									
PL → Self-esteem (direct)	0.24	0.05	0.000	0.16	0.07	0.015	0.32	0.07	0.000
Sport → Self-esteem	0.07	0.04	0.071	0.04	0.05	0.423	0.10	0.06	0.093
Indirect effect	0.02	0.01	0.075	0.01	0.02	0.427	0.03	0.02	0.101
Total effect	0.26	0.04	0.000	0.17	0.06	0.006	0.35	0.06	0.000

Standardized regression coefficient (Std B), standard error (SE), and p-values for structural equation models with aspects of emotional mental health as the outcomes. Controlled for age and sex.

**Figure 2:**
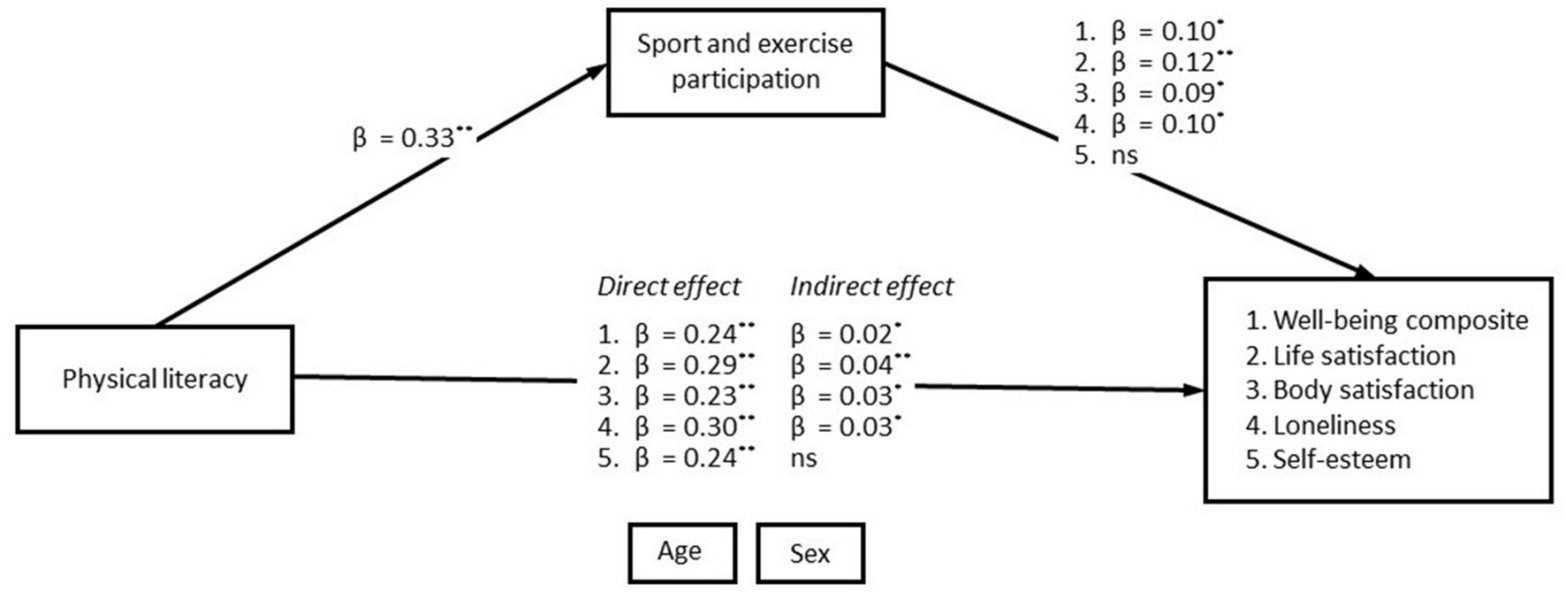
Path coefficients of the significant mediation analysis. This figure shows path coefficients of the adjusted structural equation models with aspects of wellbeing as the outcomes. All the shown parameters (β) are standardized and statistically significant. Covariation between all exogenous variables was allowed. **indicates a *p*-value under 0.01; *indicates a *p*-value under 0.05; ns, non-significant association.

The SEMs showed that PL was significant and positively associated with SEP (β = 0.33, *p* < 0.001) and with all aspects of mental health. Table 3 and Figure 2 show information about path coefficients from the five structural equation models. We observed significant positive associations between PL and all wellbeing outcomes for the total sample: wellbeing composite score (β = 0.24, *p* < 0.001), self-esteem (β = 0.24, *p* < 0.001), life satisfaction (β= 0.19, *p* < 0.001), loneliness (β = 0.23, *p* < 0.001), and body satisfaction (β = 0.30, *p* < 0.001). We found that SEP was associated with all aspects of wellbeing except for self-esteem, and only partly and to a small extent mediated the association between PL and the wellbeing composite score (indirect effect: β = 0.03, *p* < 0.001), life-satisfaction (indirect effect: β = 0.04, *p* < 0.001), loneliness (indirect effect: β = 0.03, *p* < 0.05), and body-satisfaction (indirect effect: β = 0.03, *p* < 0.05).

### Sex differences in the associations

The SEMs conducted separately for boys and girls showed that PL was found to be significantly associated with all wellbeing outcomes for both sexes, with β-coefficients ranging from 0.10 to 0.23 among boys and 0.27 to 0.36 among girls. In boys, SEP was associated with all wellbeing outcomes except self-esteem, with β-coefficients between 0.09 and 0.13. In girls, SEP only correlated with the wellbeing composite score (β = 0.10, *p* < 0.05) and life satisfaction (β = 0.14, *p* < 0.01).

We observed higher β-coefficients for the direct association between PL and wellbeing measures among girls compared to boys in all models (boys/girls)—wellbeing composite score: β = 0.16 **/** β = 0.31; life satisfaction: β = 0.10 / β = 0.29; loneliness: β = 0.16 / β = 0.29; body satisfaction: β = 0.23 / β = 0.36; and self-esteem: β = 0.16 / β = 0.33. Among boys, we observed a significant association between SEP and the wellbeing composite score (β = 0.09, *p* = 0.011), life satisfaction (β = 0.10, *p* = 0.000), loneliness (β = 0.10, *p* = 0.050), and body satisfaction (β = 0.13, *p* = 0.014) but no significant association with self-esteem. Among girls, SEP was significantly associated with the wellbeing composite score (β = 0.10, *p* = 0.016) and life satisfaction (β = 0.14, *p* = 0.001) but not with the other aspects.

## Discussion

The results of this study indicate that PL is positively associated with SEP. In the total sample, we observed an association b between PL and SEP, with a β-value of 0.33. This finding is in accordance with previous studies of cross-sectional design. Choi et al. (39) observed an adjusted association between self-reported PL and self-reported time spent in physical activities among 1945 Chinese adolescents (12–18 years of age) with a β-value of 0.23 (39), Coyne et al. (56) observed an adjusted association between PL and pedometer measured physical activity among 1,000 Canadian children (8–12 years of age) with a β-value of 0.18 (56), Melby et al. (33) found an adjusted association between PL and accelerometer measured physical activity among 647 Danish children (7–13 years of age) with a β-value of 0.39 (33), Yli-Piipari et al. (57) found that physical literacy explained 29% of their overall physical activity participation among 450 Finnish 11-year-old children (57), and, in a sample of 2,879 Canadian children (8–12 years of age), Belanger et al. (58) found that children scoring above the recommended levels of PL had higher odds of meeting physical activity guidelines (58).

The results of this study indicate that PL is positively associated with important aspects of adolescent's wellbeing. In the total sample, we observed an association between PL and emotional and social wellbeing, with β-values ranging from 0.23 to 0.30. This result is in line with previous studies. A study by Jefferies et al. (59) found an unadjusted association between PL and resilience among 227 Canadian children (9–12 years of age) with a β-value of 0.21, a study by Caldwell et al. (60) observed a positive association between PL and health-related quality of life among 222 Canadian children (mean age 10.7 years), a study by Blain et al. (32) found an unadjusted associations between PL and positive and negative affect among 187 young adolescents (mean age 12.8 years) with β-values of −0.25 and 0.38 (*p* < 0.05), and a study by Melby et al. (33) found adjusted associations between PL and four aspects of wellbeing among 647 Danish children (7–13 years of age) with β-values of 0.21–0.38. However, only few studies have investigated the association between PL and wellbeing outcomes.

Stratifying the sample by sex, we observed more pronounced associations between PL and wellbeing among girls compared to boys, with approximately double-sized β-values. The transition into adolescence is a vulnerable period (25), and girls may be particularly vulnerable to developing mental health issues (1, 61). The strong relationship between PL and emotional and social wellbeing among girls is therefore noteworthy, as it indicates that PL could potentially mitigate or reduce mental health issues among adolescents, especially amongst girls.

To our knowledge, this is the first study to investigate sex differences in the association between PL and wellbeing. Previous studies have found sex differences in the associations between SEP and wellbeing, reporting that girls have greater benefits compared to boys, especially in team sports (62, 63). However, in this study, among girls, we only observed associations between SEP and loneliness and the wellbeing composite score, which means that, when including PL in the models, SEP's relation to adolescent girls' body satisfaction, loneliness, and self-esteem were not significant. Further, β-values of the associations between PL and wellbeing outcomes were greater than those of SEP and wellbeing. In sum, the results of this study indicate that PL is more important for adolescent girls' emotional and social wellbeing than SEP. Among boys, SEP was associated with all aspects of wellbeing except self-esteem, and the β-values of the associations between PL and the wellbeing outcomes were similar (i.e., equal or a little higher) to those of SEP and wellbeing. These findings suggest that both PL and SEP are important for adolescent boys' emotional and social wellbeing.

The minimal or non-significant indirect effects of SEP on the association between PL and wellbeing demonstrate that PL is more relevant to adolescent's wellbeing than SEP. Since previous studies have found positive associations between SEP and emotional wellbeing (64–66), we wonder if the type of sport or exercise contributes to whether or not participation impacts wellbeing positively, as demonstrated in other studies that have shown that traditional team sports have a higher impact on wellbeing outcomes compared to self-organized exercise or individual sports (62, 63). This could also explain why we observed a stronger relationship between SEP and wellbeing among boys compared to girls, as boys more commonly engage in team sports, while girls engage more commonly in self-organized exercise (41). This could also explain why previous studies observed mixed findings, such as the study of Caldwell et al. (60), that found no mediating effect of accelerometer-measured physical activity on the association of PL and health-related quality of life (60) and the study of Melby et al. (33) that found a mediating effect of physical activity in the relationship between PL and one out of five investigated aspects of wellbeing (33).

The observed positive associations between PL and wellbeing could also be understood and explained through the lens of self-determination theory (34). Higher PL increases the possibility that one's basic psychological needs are satisfied in terms of experiencing competence during SEP and, accordingly, wellbeing in the activity (67). Similarly, an individual with low PL will be more likely to experience competence frustration, which will accordingly have a negative effect on their sense of wellbeing in the activity (68, 69). According to basic psychological needs theory, which has been formulated and supported in self-determination theory research, only engagement in SEP that fosters need satisfaction will positively impact overall wellbeing. Thus, SEP's impact on wellbeing strongly depends on how the SEP is delivered. This might explain why sport-based interventions exhibit mixed effects on mental health (70). One study found that the association between levels of physical activity and overall wellbeing in children was mediated by their perception of the three basic psychological needs (autonomy, competence, and relatedness) in physical activity environments (71). This result supports our theoretical assumption, outlined in the background section—namely, that wellbeing in contexts of physical activity (i.e., experiencing satisfaction of basic psychological needs) can transfer to other contexts and, ultimately, to the global level (35, 36).

Collectively, the results from this study suggest that it is critical to identify how SEP and physical activities are delivered in a way that fosters wellbeing. In this regard, supported by the observed positive association between PL and mental health in this study, it is useful to take a PL pedagogical perspective, supported by principles of the basic psychological need satisfaction, when delivering sport- or physical-activity-based interventions to increase adolescents' emotional and social wellbeing. Emerging evidence on interventions, driven by the theory of PL and aimed at increasing participation in physical activities in children up to young adulthood, has shown promise in this regard (72–74).

### Implications for practice, policy, and research

First, this study contributes to the limited evidence on the association between PL and health, supporting the assumption that PL is important for adolescents' participation in physical activities and their wellbeing. Secondly, the finding that PL has a more significant effect than SEP suggests that focus should be directed away from the current narrow focus on increasing the amount and intensity of physical activity here and now. Instead, the focus should be on supporting the development of the prerequisites for physical activity participation, i.e., the elements of PL. This so-called PL perspective seems to be more advantageous for long-term physical and mental health, including a lifelong engagement in physical activities. Those working with the physical activity of children and adolescents (e.g., physical education teachers, sport coaches, parents, and school principals) should consider how to support the development of PL by considering all of its elements and should accordingly avoid hindering one or more of these said elements. One way to achieve this is to provide appropriate challenges and demand that matches the level of participants' competence to enable experiences of need satisfaction (i.e., the need for competence), resulting in a sense of mastery and contextual wellbeing and hence autonomous motivation and confidence. Creating a task-solving/-learning environment for physical activity contexts—instead of a result-oriented or competitive environment has shown to be an effective way to this and has also shown to be beneficial for the participants' contextual wellbeing and autonomous motivation, as well as the coaches/teachers' facility of a social learning climate with a high degree of autonomy (75, 76).

Policy makers should consider including a PL perspective in addition to national guidelines on physical activity so that they contain recommendations on how best to foster the PL elements of motivation, confidence, physical competences, and knowledge and understanding that enables children and adolescents to engage in physical activities.

### Strength and limitations

A clear strength of this study was its use of a large and randomly recruited sample. It was not possible to check for representability in terms of socio-economic status in the sample of 7–15-year olds, or control for socio-economic status, which should be considered a limitation, as most health problems follow a social gradient (77, 78).

The single-item indicators of three of the aspects of wellbeing (life satisfaction, body satisfaction, and loneliness) reduced the reliability of these latent measures compared to multi-item measures. However, some of these measures have been used in similar samples and have been validated against commonly used multiple-item scales (19). Nevertheless, using the five different aspects of wellbeing added valuable information to the investigated relationships.

There was a limitation connected to the self-reported measures of this study. First, SEP was measured with a single item that prompted for the frequency of generic SEP. Secondly, self-reported measures of children's and adolescents' physical activities, such as SEP, may be considered less reliable compared to objective measures (e.g., accelerometery). Thirdly, PL was measured with a newly developed 21-item questionnaire, the MyPL questionnaire, which assessed motivation, confidence, and physical competences connected to various disciplines and contexts. The self-assessment of one's physical competences should especially be considered a limitation compared to studies using more objective direct measures/tests. On the other hand, we consider physical-activity-environment-specific prompting in the MyPL questionnaire a strength, as it deals with challenges connected to generically asking about motivation and confidence, making it further in line with the theory of PL (30).

The main limitation was the cross-sectional design, which presented vagueness about the direction of the investigated associations and put a restrain on making claims about causality. Future research should investigate the associations between PL, physical activity/SEP, and wellbeing using longitudinal and experimental designs and control for socioeconomic status.

## Conclusion

This study expands on the scarce evidence on PL's association with health. The study brings novel knowledge on the association between adolescents' PL, SEP, and emotional and social wellbeing and the mediating role of SEP in the association between PL and wellbeing. In accordance with our hypothesis, we found that PL was positively associated with SEP and all investigated aspects of emotional and social wellbeing. We found stronger associations between PL and emotional and social wellbeing among girls compared to boys, indicating that PL is particularly beneficial for adolescent girls' wellbeing. We found mixed results on the mediating role of SEP in the association between PL and the five aspects of emotional and social wellbeing. Results from this study indicate that PL likely contribute to adolescents' emotional and social wellbeing beyond its association with SEP. Implications of these results suggest focussing on supporting children's and adolescents' prerequisites for physical activity participation (i.e., the elements of PL), instead of the narrow focus on cumulative physical activity (i.e., amount and intensity).

## Data availability statement

The datasets presented in this study can be found in online repositories. The names of the repository/repositories and accession number(s) can be found at: The dataset has been submitted to the National Archives with the serial number: FD.50354.

## Ethics statement

According to a recent Danish legislation of The Danish Data Protection Agency, it is no longer required to collect consent and register the research project to the data review Centre, when the objectives of the research is in society's interest (i.e., to improve society) (79). Thus, this survey did not need to apply or register for ethical approval at the Center of data review. All procedures and handling of data were carried out based on this legislation.

## Author contributions

The study was conceptualized and manuscript was drafted by PM, PE, PB, and GN. Data management were conducted by PM and PE. All authors revised and approved the final manuscript.
