# Supplementary material for: Cross-sectional associations between adolescents' physical literacy, sport and exercise participation, and wellbeing
Source: Front Public Health. 2023 Feb 28;10:1054482. doi: 10.3389/fpubh.2022.1054482 (PMC10011712; doi:10.3389/fpubh.2022.1054482)
Supplement: Supplementary file 1 [file Data_Sheet_1.PDF]

Appendix 1, Table 1. Sample 1 (6-13 years)

	Item	Scale
**The affective domain**		
Video explanations e.g., of ball games, and an oral introduction of how to answer the questions.<br>There are two questions per context (e.g., ball games): one regarding one's motivation and one regarding one's self-confidence.		
Ball motivation	How fun is it to play with a ball?	Smiley face scale: 3-point Likert scale illustrated by smiley faces. A green happy smiley indicating "it's very fun", a yellow neutral smiley indicating "it's neither very fun nor very boring, but in-between ", and a red unhappy smiley indicating "it's very boring"<br>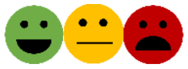
Water motivation	How fun is it to do water activities?	
Climbing motivation	How fun is it to play climbing activities?	
Running motivation	How fun is it to do running activities?	
Gymnastic motivation	How fun is it to do gymnastics and athletics?	
Skating motivation	How fun is it to do skate activities?	
Ball self-confidence	How good do you think you are at ball activities?	Star scale: 3-point Likert scale illustrated by stars. A green star indicates "I am good", a yellow indicates "I am neither good nor bad, but in between", and a red indicates "I am not that good"<br>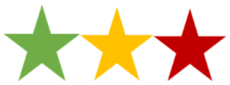
Water self-confidence	How good do you think you are at water activities?	
Climbing self-confidence	How good do you think you are at climbing activities?	
Running self-confidence	How good do you think you are at running activities?	
Gymnastic self-confidence	How good do you think you are at gymnastics and athletics?	
Skating self-confidence	How good do you think you are at skate activities?	
**The physical domain**		
Video explanation and an oral introduction on how to answer the questions. There is one question per competence.<br>The physical skills were illustrated with an age-appropriate picture and involved skipping, balance, ball, gymnastics, swimming, endurance, and strength.		
Skipping competence	Are you able to hop on one leg from side to side keeping your balance?	Symbol scale: 3-point Likert scale illustrated by three different symbols. A green symbol indicates "I am always able to", a yellow symbol indicates "I am sometimes able to", and a red symbol indicates "I am not able to"<br>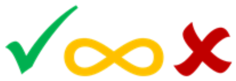
Balance competence	Are you able to jump off a tall object and land on your feet without falling?	
Ball competence	Are you able to throw a tennis ball against a wall and catch it again?	
Gymnastic competence	Are you able to do a cartwheel?	
Endurance	Are you able to run for a long time without getting tired?	
Strength	Are you able to complete ten push-ups without getting tired in your arms?	

The cognitive domain Video explanation and an oral introduction on how to answer the questions.

Transfer knowledge	In which sports activities is it important to be able to run, throw and catch?	Categorical/nominal scale with the following four options: 1) badminton, 2) volleyball, 3) handball, and 4) water polo
PA knowledge	How much time are children recommended to be physically active on daily basis? Think about activities causing short breath e.g., fast walking or running, and activities you do at home, during school hours, or in leisure time.	Categorical/nominal scale with the following four options: 1) 20-minutes, 2) 30-minutes, 3) 1 hour, and 4) 2 hours
Norm knowledge	When playing physical activities and sports, people often talk about fair play. The best description of fair play is:	Categorical/nominal scale with the following four options: 1) that one can respect rules and agreements, 2) that one tries to talk English in the PE class, 3) that one can be both a good loser and a good winner, and 4) that one does everything to win

Appendix 1, Table 2. Sample 2 (7-12 yrs.)

	Item	Scale
**The affective domain**		
A written introduction on how to answer the questions. Introduction to the five questions regarding motivation: "Some children think certain physical activities are fun, while other children think the same physical activities are boring. In the following two questions, you must indicate how fun you think different physical activities are". Introduction to the five questions regarding self-confidence: "Sometimes you might think that an activity is fun, even if you are not good at it. Other times you might be good at an activity, but think it is boring. In the following questions, you must think about how good you are at different physical activities". As for all the physical activities involved, an age-appropriate picture was used for illustrating the activity.		
Ball motivation	How fun is it to play with a ball?	5-point Likert scale ranging between "a lot of fun", "fun", "not too fun but neither too boring", "boring", and "very boring"
Water motivation	How fun is it to do water activities?	
Climbing motivation	How fun is it to play climbing activities?	
Running motivation	How fun is it to do running activities?	
Gymnastic motivation	How fun is it to do gymnastics and athletics?	
Skating motivation	How fun is it to do skate activities?	
Ball self-confidence	How good do you think you are at ball activities?	5-point Likert scale ranging between "very good", "good", "average", "fair", and "poor"
Water self-confidence	How good do you think you are at water activities?	
Climbing self-confidence	How good do you think you are at climbing activities?	
Running self-confidence	How good do you think you are at running activities?	
Gymnastic self-confidence	How good do you think you are at gymnastics and athletics?	
Skating self-confidence	How good do you think you are at skate activities?	
**The physical domain**		
A written introduction on how to answer the questions: "Think about what you actually are able to do, and not what you want to be able to do. If you are in doubt give your best guess". Additionally, the following introduction was provided: "You must answer how good you are at specific exercises that you use in different physical activities". As for all the physical domains, an age-appropriate picture was used for illustrating the physical skill.		
Skipping competence	Are you able to hop on one leg from side to side keeping your balance?	3-point Likert scale ranging between "I am always able to", "I am sometimes able to", and "I am not able to"
Balance competence	Are you able to jump off a tall object and land on your feet without falling?	
Ball competence	Are you able to throw a tennis ball against a wall and catch it again?	
Gymnastic competence	Are you able to do a cartwheel?	

Swimming competence	Are you able to swim in deep water without a floating belt or floaties?	
Endurance	How far can you run at a normal pace without stopping?<br>(if in doubt give your best guess)	Continuous categorical scale including six options:<br>"0 min.", "1-5 min.", 6-15 min.", "16-25 min.", "26-35 min.", "More than 36 min.", and "I don't know"
Strength	How long can you hold a plank, as shown in the picture?<br>(if in doubt give your best guess or give it a try)	Continuous categorical scale including six options:<br>"0 sec.", "1-19 sec.", "20-39 sec.", 40-59 sec.", 1-1½ min.", "more than 1 ½ min.", and "I don't know"
**The cognitive domain**		
A written introduction on how to answer the questions		
Transfer knowledge	In which sports activities is it important to be able to run, throw and catch?	Categorical/nominal scale with the following four options: 1) badminton, 2) volleyball, 3) handball, and 4) water polo
PA knowledge	How much time are children recommended to be physically active on daily basis? Think about activities causing short breath e.g., fast walking or running, and activities you do at home, during school hours, or in leisure time.	Categorical/nominal scale with the following four options: 1) 20-minutes, 2) 30-minutes, 3) 1 hour, and 4) 2 hours
Norm knowledge	When playing physical activities and sports, people often talk about fair play. The best description of fair play is:	Categorical/nominal scale with the following four options: 1) that one can respect rules and agreements, 2) that one tries to talk English in the PE class, 3) that one can be both a good loser and a good winner, and 4) that one does everything to win

Appendix 1, Table 3. Sample 3 (13-15 yrs.)

	Item	Scale
**The affective domain**		
A written introduction on how to answer the questions. Introduction to the five questions regarding motivation: "Some children think certain physical activities are fun, while other children think the same physical activities are boring. In the following two questions, you must indicate how fun you think different physical activities are". Introduction to the five questions regarding self-confidence: "Sometimes you might think that an activity is fun, even if you are not good at it. Other times you might be good at an activity, but think it is boring. In the following questions, you must think about how good you are at different physical activities". As for all the physical activities involved, an age-appropriate picture was used for illustrating the activity.		
Ball motivation	Indicate, how fun you think it is to play ball activities	5-point Likert scale ranging between "a lot of fun", "fun", "not too fun but neither too boring", "boring", and "very boring"
Water motivation	Indicate, how fun you think it is to swim	
Climbing motivation	Indicate, how fun you think it is to climb (e.g., tree climbing or climbing on walls)	
Running motivation	Indicate, how fun you think it is to run	
Gymnastic motivation	Indicate, how fun you think it is to do gymnastics	
Skating motivation	Indicate, how fun you think it is to skate (e.g., skateboarding or roller skating)	
Ball self-confidence	How good do you think you are at ball activities?	5-point Likert scale ranging between "very good", "good", "average", "fair", and "poor"
Water self-confidence	How good do you think you are at swimming?	
Climbing self-confidence	How good do you think you are at climbing (e.g., in trees or on a climbing wall)?	
Running self-confidence	How good do you think you are at running?	
Gymnastic self-confidence	How good do you think you are at gymnastics?	
Skating self-confidence	How good do you think you are at skating (e.g., skateboarding or roller skating)?	
**The physical domain**		
A written introduction on how to answer the questions: "Think about what you actually are able to do, and not what you want to be able to do. If you are in doubt give your best guess". Additionally, the following introduction was provided: "You must answer how good you are at specific exercises that you use in different physical activities". As for all the physical domains, an age-appropriate picture was used for illustrating the physical skill.		
Skipping competence	---	7-point Likert scale ranging from 1 to 7. The lowest score indicates “I am not able to” and the highest score indicates “I am always able to".
Balance competence	Are you able to balance on one leg with your eyes closed and your hands along the side for 10 seconds?	
Ball competence	Are you able to dribble a ball e.g., like in basketball or handball?	

Gymnastic competence	Are you able to do a handstand against a wall (without any help or support from others)?	
Swimming competence	Are you able to a headspring?	
Endurance	How far can you run at a steady pace without getting so tired that you must stop? (if in doubt give your best guess)	Continuous categorical scale including six options: "0 min.", "1-5 min.", 6-15 min.", "16-25 min.", "26-35 min.", "More than 36 min.", and "I don't know"
Strength	How long can you hold a plank, as illustrated in the picture? (if in doubt give your best guess)	Continuous categorical scale including six options: "0 sec.", "1-19 sec.", "20-39 sec.", 40-59 sec.", 1-1½ min.", "more than 1 ½ min.", and "I don't know"

The cognitive domain

A written introduction on how to answer the questions. “The next three questions are about three different topics sports. Please answer the questions based on what you know or believe. You must not get help from your parents or others.”

Transfer knowledge	In which sports activities is it important to be able to run, throw and catch?	Categorical/nominal scale with the following four options: 1) badminton, 2) volleyball, 3) handball, and 4) water polo
PA knowledge	How much time are children recommended to be physically active on daily basis? Think about activities causing short breath e.g., fast walking or running, and activities you do at home, during school hours, or in leisure time.	Categorical/nominal scale with the following four options: 1) 20-minutes, 2) 30-minutes, 3) 1 hour, and 4) 2 hours
Norm knowledge	When playing physical activities and sports, people often talk about fair play. The best description of fair play is:	Categorical/nominal scale with the following four options: 1) that one can respect rules and agreements, 2) that one tries to talk English in the PE class, 3) that one can be both a good loser and a good winner, and 4) that one does everything to win
